# Influence of Pyrolysis Temperature on the Heavy Metal Sorption Capacity of Biochar from Poultry Manure

**DOI:** 10.3390/ma14216566

**Published:** 2021-11-01

**Authors:** Jolanta Sobik-Szołtysek, Katarzyna Wystalska, Krystyna Malińska, Erik Meers

**Affiliations:** 1 Faculty of Infrastructure and Environment, Czestochowa University of Technology, 42-200 Czestochowa, Poland; jolanta.sobik-szoltysek@pcz.pl (J.S.-S.); krystyna.malinska@pcz.pl (K.M.); 2 Department of Green Chemistry and Technology, Gent University, Coupure Links 653, 9000 Gent, Belgium; erik.meers@ugent.be

**Keywords:** poultry manure, biochar, pyrolysis temperature, sorption, heavy metals, soil contamination

## Abstract

Sorption properties of various biochars have been extensively investigated by many researchers. One of the parameters that have a significant impact on sorption properties is pyrolysis temperature. This paper presents a study on the effect of pyrolysis temperature (425, 575, 725 °C) on the sorption properties of poultry-manure-derived biochar (BPM). The produced biochars, i.e., BPM425, BPM575 and BPM725, demonstrated specific properties at 425, 525 and 752 °C such as high pH (10.40, 10.65 and 12.45), high ash contents (52.07, 61.74 and 78.38%) and relatively low BET (Brunauer, Emmett and Teller) surface area (11, 17 and 19 m^2^·g^−1^). The analysis of the mineral phases of the BPMs confirmed the buffering capacity. The investigated biochars were tested for sorption of Zn, Cd and Pb in mono-, double- and triple-metal batch sorption tests. According to the obtained results, biochar produced at a temperature of 575 °C (BPM575) can function as a sufficient sorbent for the removal of Zn, Cd and Pb from a water solution. The presented results do not confirm the effect of competing metal ions on the sorption efficiency of the selected metals by the investigated biochars. Based on that, the studied biochar sorbents can be used in environments contaminated with many metals.

## 1. Introduction

One of the most important properties of biochar as a soil improver/enhancer is the potential for the sorption of organic and inorganic contaminants present in water and soil environments. Biochars can be applied as sorbents due to a number of multifunctional properties, a significant number of active sorption sites, stability and renewability, efficiency in removal of various types of contaminants, availability and low impact on the environment [1,2,3,4]. Application of biochar to soil facilitates developing strong sorption complexes, particularly in light soils with naturally weak buffering properties due to the low content of clay minerals and soil humus.

Under a high temperature during pyrolysis of organic waste into biochar, many active functional groups—both electron donors and acceptors—are created. Therefore, the surface of biochar is equipped with areas with acidic and alkaline properties which demonstrate different affinities to bind water molecules [5,6]. Due to this phenomenon, biochar reacts with both organic and mineral constituents of soil and thus demonstrates the ability to form mineral and organic complexes and occlude minerals [7,8]. This particular property determines potential applications of biochar to remove contaminants and reduce the bioavailability of many organic substances (e.g., PHAs, PCBs), plant protection chemicals and trace elements, including heavy metals, to plants [9,10,11].

Biochar sorption efficiency of various organic and inorganic contaminants depends on the temperature during pyrolysis of thermal conversion of waste into biochar. The increase in pyrolysis temperature results in the increase in specific surface area and porosity of biochar [12]. High pyrolysis temperatures allow the production of biochars that are efficient in the sorption of organic contaminants due to a higher surface area, microporosity and hydrophobicity [13,14]. Biochars produced in lower pyrolysis temperatures are more efficient in the removal of inorganic contaminants or polar organic contaminants.

The analysis of the effects of pyrolysis temperature on the properties of the produced biochar and its functionalities as an efficient sorbent [15] allowed the conclusion that temperature also has an effect on the adsorption mechanisms. Biochars produced at lower temperatures usually adsorb inorganic contaminants through electrostatic interactions, ion exchange and polarization-selective interactions. However, in the case of biochars produced at a high temperature filling the pores, hydrophobic interactions and precipitation are considered as predominant mechanisms.

Depending on the substrates and parameters of the production process, a typical particle size of a commercially available biochar ranges from a millimeter to a centimeter [16]. However, commercially available biochars are composed of particles with diameters smaller than a few tens of micrometers. This leads to an increase in surface area. As reported by Yang et al., the colloidal particle size of biochar ranges from 0.1 to 53 µm, and its ratio is usually 1–2% by weight [17,18]. It has to be pointed out that a higher content of ash in biochars could limit the development of the specific surface area (BET) and the total porosity due to the inhibition of micropore formation with simultaneous development of mesopores [19].

Sorption of metals in the course of electrostatic attraction, ion exchange, surface complexation and precipitation of metals through releasing soluble ions occurs on biochar due to the presence of mineral constituents. Metal ions are heavily adsorbed on specific, active sites of biochar which comprise phenolic and carboxylic functional groups present on the surface [20]. The basic mechanisms for the stabilization of heavy metals by biochars include the following [21,22,23]:

- Metal ion exchange with Ca^2+^ and Mg^2+^ cations and also other cations bound with biochar;
- Metal complexation by functional groups and inner complexation by hydroxyl groups;
- Mechanisms based on electrostatic interactions;
- Physical adsorption and precipitation on biochar surface solid particles.

Due to specific properties—in particular, a porous structure and high availability of substrates, including a diverse group of biodegradable waste for biochar production—the application of biochar as a material for the production of sorbents for heavy metals is in line with the rules for sustainable development, is economically justified and could bring a number of benefits to the environment. Biochars as sorbents produced from waste could be an alternative for activated carbons as they demonstrate similar properties [24]. What is more, as opposed to activated carbon, biochar contains a non-carbon fraction that can additionally react with contaminants present in soil enhancing the efficiency of sorption [25].

In reference to the above-mentioned properties of biochars, the overall goal of this work was to analyze the effect of pyrolysis temperature on the sorption properties of biochars produced from poultry manure toward the sorption of ions of selected heavy metals. The novelty of this work lies in analyzing the effect of the presence of competing ions on the sorption of the investigated heavy metals in mono-, double- and triple-metal sorption experiments. We tested the hypothesis that competing metal ions can have an effect on the sorption of selected metals in multi-metal combinations. Metals with higher affinity can replace metals with lower affinity when metals are competing for sorption sites [26].

The scope of this study included: (1) production of biochar from poultry manure through pyrolysis under selected temperatures in a laboratory pyrolysis reactor (425, 575 and 725 °C), (2) physicochemical and chemical analysis of poultry manure derived biochar and (3) batch sorption tests of Zn, Cd and Pb in mono-, double- and triple-metal sorption experiments.

The presented results contribute to the development of the state of the art of the effects of pyrolysis temperature on the sorption properties of biochar produced from poultry manure. Biochar as a sorbent can be used for removing metals from a liquid solution or when added to soil as a soil improver to stabilize metals in soil.

## 2. Materials and Methods

### 2.1. Substrates for Biochar Production

Biochar was produced from poultry manure (indicated throughout this paper as PM) sampled from a poultry farm located in Southern Poland with 120,000 laying hens and 1 mln broilers in cage system in every production cycle (personal communications). Poultry is fed with customized feed obtained from cereal grains delivered by local farmers. Fresh poultry manure was sampled, transported to the lab, dried in the oven at 105 °C and crushed in a laboratory crusher (A-11 Basic, IKA, Freiburg, Germany). Sampled poultry manure (in 3 replications) was analyzed for pH, moisture content, ash, total carbon, total nitrogen and phosphorous. These properties are presented in Table 1.

### 2.2. Parameters of the Pyrolysis Process

The substrates were subjected to thermal conversion in a pyrolysis reactor (PRW-S100x780/11) in nitrogen atmosphere (5 L min^−1^). The pyrolysis reactor was designed and manufactured by the Polish company Czylok for laboratory use at the Czestochowa University of Technology (Poland). The heating temperatures of the substrate were 425, 575 and 725 °C, and the heating time of samples was 120 min. The retention time was 60 min. After the pyrolysis process was completed, the samples were left in the reactor until they reached room temperature. Biochar samples were stored in tightly closed containers at room temperature. The biochars produced in the pyrolysis of PM at selected temperatures are referred to as BPM425, BPM575 and BPM725 throughout this paper.

### 2.3. Physicochemical and Physical Analysis

Poultry manure and the obtained biochars were analyzed for moisture content (by oven drying at 105 °C) and ash in accordance with PN-EN ISO 18122:2016-01 [27]. These materials were analyzed for the total carbon content (by multi N/C, Analytik Jena—high-temperature incineration with detection IR, Jena, Germany) and Kjeldahl nitrogen content in accordance with PN-EN 16169:2012 [28]. The pH measurement was carried out by placing 5 g of the sample into three individual beakers and then adding distilled water to each of them (50 mL). The samples were shaken for 10 min and then infiltrated. The pH was measured by a standard pH meter. Biochars derived at selected temperatures were analyzed for the elemental composition, total organic carbon content and surface area. The CHNS elemental analysis was performed with the Thermo Scientific™ FLASH 2000 (Waltham, MA, USA) method of dynamic incineration (3–4 independent incineration). The total organic carbon content was determined according to the Polish standard PN-ISO 10694:2002 [29]. The BET surface area was determined through nitrogen gas sorption by the ASAP 2020 Plus analyzer manufactured by Micromeritics (Atlanta, GA, USA).

The investigated biochars (BPM425, BPM575, BPM725) were subjected to XRD analyses which were performed on powdered biochar samples using a PANalytical X’Pert Pro MPD (multipurpose diffractometer) powered by a Philips PW3040/60 X-ray generator (Malvern Panalytical B.V., Almelo, The Netherlands) and fitted with a 1D silicon strip detector (X’Celerator). The measurements were performed using Co Kα-radiation with a wavelength of 0.1789010 nm, an acceleration voltage of 40 kV, a current of 40 mA and 0.02° 2θ step sizes (some samples with 0.02° 2θ step size) between the angles of 3 and 90° 2θ and a 400 s measurement time per step. The data obtained were processed using HighScore+ software (version 4.9) and the ICSD database and PDF4+ ICDD database.

Chemical analyses of biochar for the selected elements (P, K, Ca, Mg, Hg, Pb, Cd, Cr, Cu, Ni, Zn) were performed with the ICP method according to the standard. The cation exchange capacity (CEC) was determined according to the following protocol: a biochar sample (1.25 g) was transferred to a bottle of 100 cm^3^ and treated with 50 cm^3^ of 1 M NH_4_Cl extraction solution. The bottles with the suspension were shaken in a laboratory shaker (40 rotation/min) for 2 h. Then, the suspension was filtered through a hard filter (Whatman, UK). The concentration of elements (Ca, M, K, Na) was determined with the spectrophotometric method (ICP-OES Thermo Elemental IRIS Intrepid II XSP DUO). All tests were run in triplicates, and the results were expressed as the average values.

### 2.4. Batch Sorption Tests for Zn, Cd and Pb

Prior to the study on sorption capacity with batch tests, each type of produced biochar was milled and passed through a 500 μm sieve. Biochar samples were placed in glass bottles and filled with the prepared solution with the investigated metal ions. The bottles with the suspension were shaken in a laboratory shaker (150 rotation/min) for 24 h. Then, the suspension was filtered through a hard filter (Waterman) and a syringe filter (45 µm). The concentration of the investigated metals (Zn, Pb, Cd) in the solutions was determined with the spectrophotometric method (ICP-OES Thermo Elemental IRIS Intrepid II XSP DUO). The concentration of heavy metal ions in the initial solution was obtained by dissolving the suitable mass of the following salts: ZnCl_2_, CdCl_2_·2.5 H_2_O and Pb(NO_3_)_2_ in deionized water. The salts were purchased from the ChemPur supplier (Piekary Śląskie, Poland). To assure the equilibrium of ion force, the pH of the obtained solutions was maintained at 4.0 with NaOH or HCl, respectively.

Batch sorption tests were performed in 2 phases (Phase I and Phase II) described in detail in Table 2. The concentrations of metals in the solutions were selected based on the literature references and studies carried out by other researchers. The range of the concentrations of metals in the solution which was tested in the second phase of batch sorption tests was limited to 3 values due to the lack of significant differences in mono-metal sorption at the lowest and highest concentrations. In the present study, the ratio of biochar to liquid in Phase I was 1:10 (10 g of biochar to 100 mL of the solution) in the range reported in the literature, e.g., by Van Poucke et al. [30]. In Phase II, we used also a smaller dose of biochar (5 g of biochar in 100 mL of the solution) in a ratio of 1:20. All sorption experiments were run in duplicates.

The removal (R) of the investigated metal from the solution was calculated according to the following formula:

(1) $$R=\frac{C_{1}-C_{2}}{C_{1}}100\;\left(\%\right)$$

where:

C_1_—the initial metal concentration in the solution, mg·L^−1^.
C_2_—the final metal concentration in the solution, mg·L^−1^.

Sorption capacity of biochar was calculated according to the following formula:

(2) $$A=\frac{C_{1}-C_{2}}{m}\cdot V\;\left(\mathrm{mg}\cdot g^{-1}\right)$$

where:

A—adsorption, mg·g^−1^,
V—the volume of the solution, L,
m—the weight of biochar, g.

### 2.5. Statistical Analysis

The statistical analysis was performed by the IBM SPSS Statistics 26. The significance level was α = 0.05. The aim of the statistical analysis was to verify the relationship of biochar production temperature and sorption capacity toward Zn, Cd and Pb in mono- and multi-metal experiments. Combination of metals (4 types of metals) and pyrolysis temperature (3 values of temperature) was an independent variable whereas sorption of Zn, Cd and Pb was a dependent variable. The experiment results were tested with two-way ANOVA.

## 3. Results

### 3.1. Biochar Production Yield

Biochars from poultry manure were produced at 3 different pyrolysis temperatures: 425, 575 and 725 °C. The yield of biochar for these temperatures was as follows: 52.79, 43.94 and 40.16%, respectively. With the increase in pyrolysis temperature from 425 to 725 °C, the biochar yield decreased by 12.63%. Similar results for thermal conversion of poultry manure into biochar were obtained by Song and Guo [31], Novak et al. [32], Srinivasan et al. [33] and Bavariani et al. [34]. The biochar production yield also depends on the type, origin and composition of the substrate. Some researchers used poultry manure mixed with bedding whereas other researchers used poultry manure.

### 3.2. Chemical Composition of Biochars

The produced biochars were subjected to physicochemical analysis (Table 3, Table 4, Table 5 and Table 6). The content of Ca in biochars was in the range of 12.7–18.10% and was higher than in biochars produced at higher temperatures (Table 3). Other researchers reported that the content of Ca in the investigated biochars was in the range of 7.17–9.40% [31]. They also observed an increase in the Ca content with an increase in pyrolysis temperature. A similar tendency was observed for Mg which was in the range of 1.32–1.5% in the produced biochars. According to Zhang et al. [26], the presence of Ca^2+^ and Mg^2+^ could have a predominant effect on the sorption of other ions by biochar. However, the content of Ca^2+^ and Mg^2+^ in biochars investigated by these authors [26] was significantly higher (84.91–173.03 mg·kg^−1^ and 3.31–6.93 mg·kg^−1^, respectively) than in the biochars investigated in this study. This was due to the fact that a different substrate (*Medulla tetrapanacis*) was used by Zhang et al. [26] to produce biochar.

The analysis of nutrients such as P and K showed that their content was in the range of 3.28–4.0% and 4.47–5.55%, respectively. Similar results were reported in the literature [31,32,34]. With the increase in the pyrolysis temperature, the content of P and K also increased. The concentration of these elements in the biochars produced at higher temperatures was usually higher than at lower temperatures. Bavariani et al. [34] reported that the content of P in biochars produced at 200 °C was 3.39% whereas, in biochars produced at 500 °C, it was 6.38%. Higher contents of P in biochar produced at 700 °C (4.28%) were also reported by Novak et al. [32]. As for biochars produced at 350 °C the content of P was 2.94%. The analysis of biochars performed by Bavariani et al. [34] demonstrated a higher content of P in biochar produced at 500 °C (1.97%) whereas, at 200 °C, the content of P was 1.04%.

The produced biochars were also tested for the concentration of the following heavy metals: Cd, Pb, Cr, Cu, Ni, Zn and Hg (Table 3). The highest Cd concentration of 1.04 mg·kg^−1^ was determined in BPM425. For biochars produced at higher temperatures, lower concentrations of Cd were determined. For example, for the BPM725 biochar, the concentration of Cd was <0.300 mg·kg^−1^. The concentration of Pb was in the range of 8.77–10.20 mg·kg^−1^ whereas, for Cr, it was in the range of 22.9–27.8 mg·kg^−1^. The concentration of Ni was in the range of 20.5–30.9 mg·kg^−1^. The Hg concentration in the investigated biochars was less than 0.0056 mg·kg^−1^. Srinivasan et al. [33] also investigated the concentration of heavy metals in biochar produced from poultry manure. The researchers reported the following concentrations: Zn 195.4 mg·kg^−1^, Cu 24.77 mg·kg^−1^, Cr 12.25 mg·kg^−1^, Cd 0.28 mg·kg^−1^, Pb 2.31 mg·kg^−1^ and Hg 0.02 mg·kg^−1^. A comparison of the results from this study and the current study’s results shows that the concentrations of Zn, Cu, Cr, Cd and Pb were lower, particularly in the case of the concentrations of Cu and Zn reported by Srinivasan et al. [33]. However, Bavariani et al. [31] reported that the concentration of Cu in the biochars produced at the temperatures of 200–500 °C was in the range of 99.6–191 mg·kg^−1^ whereas the concentration of Zn was in the range of 565–1023 mg·kg^−1^.

The analysis of elemental composition (Table 4) for the investigated biochars showed that the content of C was 37%. Higher content of C is typical for plant-derived biochars [33]. The content of H was in the range of 1.0–2.16% and decreased with the increase in pyrolysis temperature. A similar tendency was observed for nitrogen which decreased from 4.81% to 2.5%. The content of sulfur in the investigated biochars slightly increased with the increase in pyrolysis temperature. This was also observed by Song and Guo [31]. The obtained results from the elemental analysis allowed the calculation of the following ratios H/C, O/C and (N + O)/C which are used to characterize the properties of biochars. The ratio of H/C of the investigated biochars significantly decreased with the increase in pyrolysis temperature which indicates an increase in aromaticity [19]. A similar tendency was observed for the ratios of O/C and (N + O)/C which can indicate a drop in polarity and decrease in the number of surface functional groups containing oxygen [19,26].

### 3.3. Phase Analysis of Biochars

**Figure 1:**
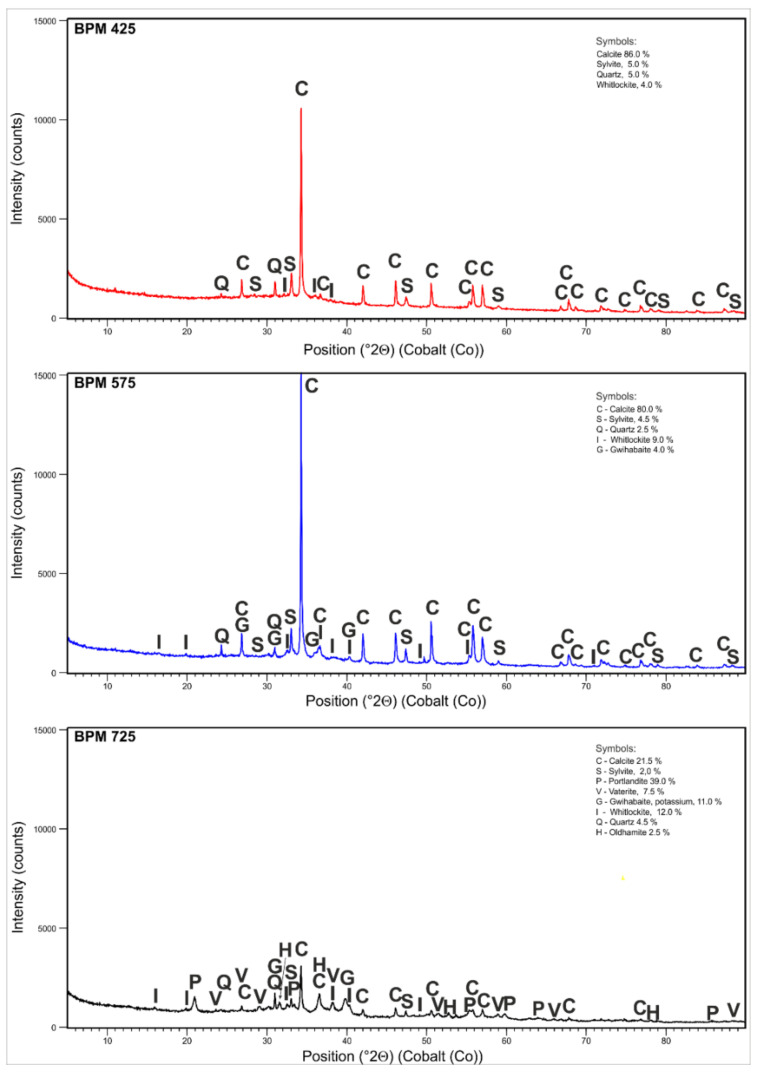
X-ray diffraction analysis of the BPM425, BPM575 and BPM725 biochars.

Due to the fact that biochars produced from poultry manure demonstrated significant contents of mineral substances, the investigated poultry-manure-derived biochars were also subjected to the phase analysis with XRD method. The obtained results are presented in Figure 1 and Table 5. In the BPM425 and BPM575 biochars, calcite is the predominating mineral phase whereas, in the BPM725 biochar, the content of calcite is 4-fold lower. This is due to the thermal decomposition of CaCO_3_ and the presence of a new carbonate phase which is vaterite. The presence of numerous mineral phases containing Ca is confirmed by the chemical composition of the investigated biochars (Table 3). The presence of mineral phases bound with phosphorous is observed in all biochars in the form of phosphates. The highest content of phosphates in the form of whitlockite is detected in the BPM725 biochar which was confirmed by the content of phosphorous in this biochar (Table 3). The presence of whitlockite as a mineral phase in biochars produced from animal-derived biomass was also confirmed by Cao and Harris [35]. Potassium was bound in the form of sylvite in all biochars. However, in BPM575 and BPM725 biochars, potassium is also present as a mineral phase, i.e., gwihabaite.

**Table 5:** The main mineral phases in the investigated biochars.

Ref. Code	Mineral Name	Chemical Formula	SemiQuant, %		
			BPM425	BPM575	BPM725
01-075-6049	Calcite	Ca(CO_3_)	86.0	-	-
01-086-2334	Calcite	CaCO_3_	-	80.0	21.5
00-041-1476	Sylvite, syn.	KCl	5.0	-	2.0
04-007-9713	Sylvite, syn.	KCl	-	4.5	-
01-087-2096	Quartz, syn.	SiO_2_	5.0	-	-
01-075-8320	Quartz	SiO_2_	-	2.5	4.5
01-076-8438	Whitlockite, syn.	Ca_3_(PO_4_)_2_	4.0	-	-
98-000-0800	Whitlockite	Ca_2.89_ Mg_0.11_ (PO_4_)_2_		9.0	12.0
00-050-1566	Gwihabaite	(NH_4_, K) NO_3_	-	4.0	-
04-014-2252	Gwihabaite, potassium syn.	K_0.366_ (NH_4_)_0.634_ (NO_3_)	-	-	11.0
00-024-0030	Vaterite, syn.	CaCO_3_	-	-	7.5
98-004-6287	Portlandite	Ca(OH)_2_	-	-	39.0
98-001-7476	Oldhamite	CaS	-	-	2.5

### 3.4. Selected Physicochemical Properties of the Investigated Biochars

**Figure 2:**
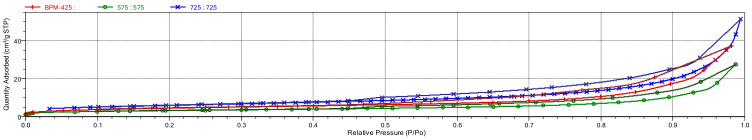
The adsorption–desorption isotherms BET.

The obtained physicochemical properties of biochars are presented in Table 4. All types of biochars demonstrated alkaline reactions (pH > 10) which increased with the increase in the pyrolysis temperature. The obtained pH values can indicate a significant potential of these biochars toward the sorption of positively charged ions of heavy metals [26]. The content of ash in biochar was high and increased with the pyrolysis temperature reaching values in the range of 52.07–78.38%. High contents of ash are usually detected in biochars derived from excreta, e.g., sewage sludge and poultry manure [32,36], and thus can affect the specific surface area of biochars. According to Novak et al. [32], inorganic constituents of ash can block the pores and thus reduce the specific surface area. However, the mineral fraction of biochars can play an important role in the process of sorption of heavy metal ions. This is even more important than the biochar structure [26]. The BET surface area of the investigated biochars reached the maximum value (19 m^2^·g^−1^) for the BPM725 biochar which is high in comparison to the results obtained by other researchers. For example, in the work Zhao et al. [37], the specific surface area of biochar produced at 550 °C was 7.09 m^2^·g^−1^. The adsorption–desorption isotherms for the investigated biochars are presented in Figure 2.

Analyzing the shape of these isotherms following the IUPAC classification (International Union of Pure and Applied Chemistry), they can be classified into type II which describes sorbents containing macropores or with a flat surface. In addition, according to the IUPAC classification, the shape of the hysteresis loop indicates the H3 type which is characterized by the presence of non-rigid plate aggregates. The structure—as reported by Zhang et al. [26]—can facilitate the sorption of heavy metal ions. The literature reports [22] that a higher temperature usually leads to the formation of larger-size pores which increases the surface and porosity of sorbents (Table 6).

**Table 6:** Selected properties of biochar.

Type of Biochar	pH	M	Ash	BET	Porosity	CEC	TOC
		%		m^2^·g^−1^	cm^3^·g^−1^	cmol(+)·kg^−1^	% d.m.
BPM425	10.40	1.95 ± 0.11	52.07 ± 1.07	12	0.037 ^a^	31.9	35.15
BPM575	10.65	2.30 ± 0.28	61.74 ± 1.59	17	0.058 ^b^	118.9	29.00
BPM725	12.45	0.10 ± 0.04	78.38 ± 0.56	19	0.060 ^c^	386.3	31.62

^a^ total pore volume, determined at P/P_0_ = 0.9776, ^b^ total pore volume, determined at P/P_0_ = 0.9815, ^c^ total pore volume, determined at P/P_0_ = 0.9754.

As indicated by Li et al. [19], biochars from poultry manure are characterized by higher polarity (higher ratio of O/C) in comparison to biochars from other substrates. These authors pointed out that a higher content of ash can secure polar functional groups of organic matter from the removal during pyrolysis. The ratio of O/C of the investigated biochars was in the range of 0.26–0.34 and gradually decreased with the increase in the temperature of biochar production. The value of the O/C ratio of the investigated biochar was higher than the values reported by Srinivasan et al. [33] and Novak et al. [32]. In addition, Li et al. [19] analyzed biochars with the ratio of O/C higher than the investigated poultry-manure-derived biochars which was about 0.46–0.48.

The cation exchange capacity (CEC) of the investigated biochars ranged from 31.9 to 386.3 cmol(+)·kg^−1^ and increased with the pyrolysis temperature. This tendency was confirmed by other researchers. Bavariani et al. [34] studied the cation exchange capacity (CEC) of biochars produced from pyrolysis of poultry manure which was in the range of 58.0–86.5 cmol·kg^−1^. According to Meszaros et al. [38], the presence of Ca, Na and K facilitated the formation of oxygen functional groups on the surface of biochars, thus resulting in higher values of CEC. This is confirmed by the obtained results (Table 3) which demonstrate that the contents of these elements increased with the increase in the pyrolysis temperature and thus increased the value of CEC.

The obtained TOC values were in the range of 29.0–39.78%. Low contents of organic carbon in the investigated biochars—which is of great importance from the perspective of agricultural applications—do not exclude them from other applications, e.g., as sorbents for soil contaminants.

### 3.5. Batch Sorption Tests

#### 3.5.1. Mono-Metal Sorption Test—Phase I

**Figure 3:**
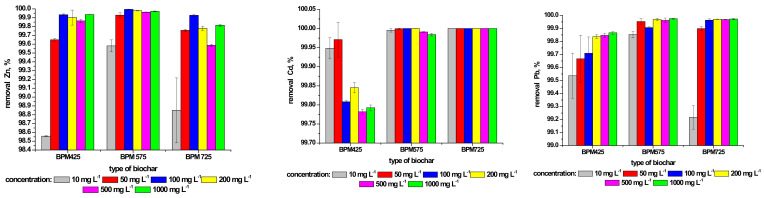
The removal of Zn, Cd and Pb (%) determined for the produced biochars in relation to the initial concentration of selected metal in the solution.

The first step of this study included the analysis of the capability of biochars to remove metal ions from the solutions with six initial concentrations (Table 2) of each ion and in the presence of only single ions in the solutions. Sorption capability expressed as a percent of Zn, Cd and Pb removal from the solution and calculated from the formula (1) is presented in Figure 3. In all analyzed cases, the removal (%) of metal from the solution was higher than 99%. This could have resulted from the precipitation of phosphates due to a high concentration of phosphorous in BPMs at a relatively small specific surface area of biochars. The presence of carbonates and phosphates in the composition of biochars facilitates the precipitation of metals from the solutions through the formation of insoluble complexes, thus increasing sorption capability. The concentrations of these anions depended on pyrolysis temperature and time [30]. A similar phenomenon was observed by Cao and Harris [35]. They pointed out the effect of high pH values and the presence of calcite in biochars. In our study, the presence of calcite in the investigated biochars was also identified (Table 5).

Irrespectively of the type of a sorbed ion, the biochar produced at the temperature of 425 °C demonstrated lower sorption capability from other investigated biochars. The removal of Zn occurred at the highest efficiency in all tested concentrations for the BPM575 biochar. Analyzing the effect of the applied initial metal concentrations, it was observed that the highest efficiency—irrespectively of the biochar type—was achieved for the concentration of 100 mg·L^−1^ whereas the lowest efficiency was observed for the concentration of 10 mg·L^−1^. In the case of the BPM725 biochar for concentrations higher than 100 mg·L^−1^, lower efficiency was observed for the removal of metal for the remaining biochars. The efficiency of sorption of Cd ions was the highest for the BPM725 biochar. In this case, the removal of metal from the solution reached 100% (irrespectively of the initial metal concentration). The most significant decrease was observed for the concentration of 500 mg·L^−1^. Similar efficiency was observed for the BPM575 biochar. As for the BPM425, we observed the effect of the initial metal concentration on the removal efficiency. The efficiency decreased with the concentration, and the most significant decrease was observed at the concentration of 500 mg·L^−1^.

As for Pb, the lowest efficiency for metal removal was demonstrated by the BPM425 biochar. However, at concentrations higher than 50 mg·L^−1^, the removal efficiency of this metal ion from the solution increased. Similar to Zn, the removal efficiency of Pb for the concentration of 10 mg·L^−1^ was the lowest for each of the investigated biochars. Analyzing the effects of all investigated biochars, it has to be pointed out that the optimal conditions for sorption of Pb were demonstrated by the BPM575 biochar. The removal (%) of Pb after 24 h—which was investigated by Park et al. [39]—was 98.6%. These researchers stated that high concentrations of nitrogen and sulfur in this biochar could contribute to the complexation and precipitation of Pb. In addition, it has to be pointed out that these researchers used biochar with a high ratio of O/C (0.85). This indicates a significant number of polar groups. As for the investigated biochars, the ratio of O/C was lower (Table 4) which can indicate that the ion removal of this metal is governed by other mechanisms.

Cations of divalent metals demonstrate a strong tendency toward hydration in the water solution—which is dependent on the pH. Therefore, metals such as Zn, Cd and Pb demonstrate similar sorption mechanisms, i.e., cation exchange, surface complexing, precipitation and electrostatic interactions. For example, cadmium at pH > 11.0 is present in the form of a hydroxy complex. This was also reflected in the present study (Figure 3).

The removal of Cd by the BPM725 biochar irrespective of the initial concentrations was 100%. What is more, after sorption was completed, the pH of the solution was above 12.6. Park et al. [40] studied the adsorption capacity of single heavy metals by biochar produced from sesame straw at 700 °C. They found that the adsorption capacity of this biochar is as follows: Pb > Cd > Cr > Cu > Zn. In the present study, poultry-manure-derived biochar was produced at a similar temperature (725 °C) and followed the pattern Cd > Pb > Zn.

Adsorption (A) was calculated for each metal and the initial metal concentration in the solution (the biochar dose of 100 g·L^−1^) based on Formula (2). The analysis of the obtained results (Table 7) showed that the sorption capacity of Zn, Cd and Pb for the BPM425 was in the range of 0.0985–9.9935 mg·g^−1^, 0.0999–9.9793 mg·g^−1^ and 0.0995–9.9866 mg·g^−1^, respectively. Similar values were observed for the BPM575 and BPM725 biochars in the entire range of investigated concentrations of ions in the solutions. With the increase in the concentrations of the selected metal in the solution, the sorption capacity increased for all investigated biochars. This shows that these biochars still demonstrate unutilized capability for the sorption of metals. No relationship between sorption capacity for a selected metal ion and the type of biochar was observed. However, it has to be pointed out that the sorption capacity for Cd demonstrated a significantly higher value for this ion in comparison to Zn and Pb in the assumed experimental conditions. Park et al. [39] observed the opposite relationship in their study on biochar produced at 550 °C from poultry manure. The researchers explained this by the lower value of the electronegativity constant for Cd (0.69) in relation to Pb (2.33).

The obtained adsorption values in the present study (Table 7) did not exceed 10 mg·g^−1^ and were lower than the values reported by El-Banna et al. [41]. However, these researchers used biochars modified with HNO_3_ and KMnO_4_ which had an impact on the sorption potential. This indicates the need to analyze the potential of the investigated biochars for modifications to improve their sorption properties.

**Figure 4:**
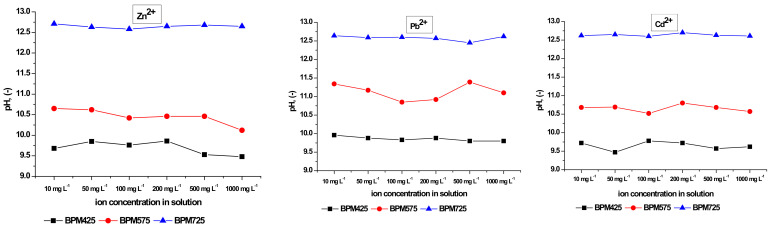
The pH values after the process of sorption was completed.

pH determined the course of the sorption process, and thus, in the presented study, it was monitored and verified. It has to be pointed out that to maintain the same ionic force, all the initial solutions—prior to the start of sorption experiments—were adjusted to pH=4. The pH values measured after the process of sorption are presented in Figure 4. Many researchers studied the relationship between the pH and the pyrolysis temperature of biochar produced from sewage sludge [42,43] and manure [44]. Jin et al. [43] reported that the increase in temperature leads to the increase in the ash content in biochar which positively correlates with its pH. Ash is considered as a factor that contributes to high values of biochar pH. This was also confirmed in the present study (Table 6). Based on the analysis of the solution pH after the sorption was completed, it can be stated that, with the increase in the pyrolysis temperature of biochars, alkalization of the solution also increases. Each investigated case showed that after sorption was completed, pH values were higher than 9.6 irrespective of the ion type and the biochar type. This is one of the reasons for the high-efficiency removal of metals which was about 99%.

#### 3.5.2. Double- and Triple-Metal Sorption Tests—Phase II

According to Park et al. [40] and Zhang et al. [26], studies on the adsorption of heavy metals by biochars in the presence of competing ions are crucial prior to analyzing adsorption in the natural environment. Park et al. [40] demonstrated that biochars from sesame straw produced at 700 °C showed the adsorption capacity for the investigated ions in the following sequence: Pb > Cu > Cr > Zn > Cd. This means that Pb is strongly adsorbed whereas Cd is weakly adsorbed. Therefore, in the second part of this study, we decided to perform batch sorption tests with the investigated biochars for the selected concentrations of metals (50, 100, 200 mg·L^−1^) in double- and triple-metal combinations (Zn, Cd, Pb) maintaining the same initial concentrations for all metals. Taking into account the economic aspect (i.e., the cost of biochar production), the experiment was performed with two doses of biochar reducing the dose in the first part of the study by half. Table 8, Table 9 and Table 10 present the removal of all three metals from the solutions containing one, two or three types of metal ions.

The obtained results demonstrate that, for all investigated biochars and at the assumed experimental conditions, the metal removal in single-, double- and triple-metal combinations was higher than 99%. We did not observe any significant effect of biochar dose or the initial metal concentration in the solution on the removal. According to the work by Zhang et al. [26], the removal of metal ions decreased with the increase of the initial concentration, in particular for the concentration of 200 mg·L^−1^, and the removal was higher with the increase in biochar dose.

The analysis of removal of selected metal ions from the solutions demonstrated a statistically significant main effect of the variable which was the type of biochar determined by the production temperature (Table 11, Table 12 and Table 13). According to the calculations, no significant main effect of the tested metal combination and no interactive effect between the type of biochar and metal combination were detected. This indicates that metal combination does not differentiate the sorption efficiency and also does not have an effect on the relationship of biochar production temperature and the sorption level. These results refer to all investigated metal ions (Zn, Cd, Pb).

Post hoc tests performed using the results of the Zn removal (%) from the solution demonstrated statistically significant differences only in the sorption effect with the use of the BPM425 and BPM575 biochars. This means that the removal (%) of Zn is higher with the use of the BPM575 biochar in comparison to the effect achieved by the BPM425. The obtained results show that the metal combination does not differentiate the sorption level of Zn. In addition, it does not have an effect on the relationship of biochar production temperature with the level of this metal. The results of the statistical analysis for Zn are presented in Table 11.

The analysis of the dependent variable, i.e., sorption expressed as the removal (5) of Cd, allowed the disclosure of a statistically significant main effect of the variable which is the temperature of biochar production. The comparison of pairs (post hoc calculations) demonstrated two statistically significant differences between the BPM425, BPM575 and BPM725 biochars. No statistically significant differences between the effect of BPM575 and BPM725 applications were observed. This means that the sorption efficiency of Cd is lower in the case of BPM425 when compared to the application of BPM575 and BPM725. At the same time, no main effect of metal combination and interactive metal combination with biochar pyrolysis temperature was reported. Therefore, metal combination does not differentiate the removal of Cd and does not have an effect on the relationship of the biochar type and sorption of this metal. Table 12 presents the results of the statistical analysis.

The calculations for the dependent variable expressed as the removal of Pb (%) also did not show any statistically significant main effect of the dependent variable which is the temperature of biochar production. The comparison of pairs (post hoc tests) for the analyzed variable indicated statistically significant differences between the effect of BPM725 and two biochars, i.e., BPM425 and BPM525. No differences between the effect of BPM425 and BPM575 application were detected. This means that the removal of Pb was higher when the BPM725 biochar was used than the BPM425 and BPM575 biochars. The results of this analysis do not reveal any statistically significant main effect for metal combination and the interactive effect of metal combination and biochar production temperature, suggesting that metal combination does not differentiate the level of Pb sorption and also does not have an effect on biochar production temperature and sorption of Pb. The detailed tests are presented in Table 13.

Comparing the results of sorption capacity (adsorption) of a selected metal ion in single, double or triple combinations (Table 14), it was observed that the application of a smaller biochar dose resulted in an increase in the sorption capacity by one magnitude for all investigated cases. The analysis of data presented in Table 14 showed that the increase in the metal concentration in the solution resulted in the increase of adsorption of all investigated biochars.

The analysis of sorption capacity of biochars toward Zn demonstrated that the presence of competing ions in the solution resulted in a slight decrease in the value of this parameter for the investigated biochar at the concentration of metal ions in the solution of 50 mg·L^−1^. At the same concentration, Cd was the most competing ion. The presence of Cd ions had an effect on the sorption of Zn by all the investigated biochars. For the concentration of 100 mg·L^−1^, despite the presence of two competing ions in the solution, a slight increase in the adsorption of Zn was observed. A similar situation occurred at 200 mg·L^−1^, however only for a smaller biochar dose because, for bigger doses, the tendency was quite the opposite. At this concentration, Cd was also a competing ion.

The obtained values of Cd adsorption at all investigated concentrations of metals in the solution indicated that the presence of competing ions increases this parameter. However, the presence of Pb in the solution results in the reduction of the adsorption of Cd.

The analysis of the adsorption of Pb showed that the presence of competing ions in the solution at the concentration of 50 mg·L^−1^ has an effect on this parameter. The presence of Zn ions contributes to the reduction of the Pb adsorption whereas the presence of Cd ions contributes to its increase. The triple-ion combination in the solution results in the highest adsorption. At the concentration of 100 mg·L^−1^, a positive effect of Zn and Cd ions present in the solution on the Pb adsorption was observed. Similar results were observed for the concentration of 200 mg·L^−1^ as well as the concentration of 50 mg·L^−1.^

Taking into account the effect of pH on the course of sorption and similarly to the first part of this study, this parameter was subjected to verification (Table 15). Based on the analysis of the obtained results, we observed similar relationships as in the first part of this study, i.e., the increase in biochar production temperature resulted in the increase in alkalization of the solutions (in each case, the initial solution showed pH = 4). Therefore, the combination of ions in the solution does not affect this parameter, and the capabilities for alkalization of the solutions are related to the content of ash in biochar.

## 4. Conclusions

The results obtained from the presented study show that biochars produced from poultry manure demonstrate potentials for the removal of selected heavy metals. It has to be highlighted that the literature reports a great number of studies on plant-derived biochars and their sorption potential. However, information on the potential of poultry-manure-derived biochars for the sorption of heavy metals is scarce. Pyrolysis temperature is a process parameter that determines the properties of biochars. Temperature has a significant effect on the pH, ash content, CEC and BET specific surface area of the investigated biochars. The analysis of sorption of metal ions in mono-, double- and triple-element batch tests showed that the type of biochar (produced at different temperatures) has an effect on the efficiency of the removal of selected metals from the solution. Statistical analysis did not confirm the effect of competing metal ions on the efficiency of the removal of the selected metal.

Based on the obtained results, the efficiency of Zn removal from the water solution is sufficient with the biochar produced at 575 °C. As for the sorption of Pb and Cd, the highest efficiencies were obtained with the biochar produced at 725 °C. However, it has to be underlined that, irrespective of the assumed experimental conditions, the application of the BPM575 biochar as a sorbent is sufficient for the removal of these metals from the solution. What is more, the costs of biochar production are expected to be lower due to lower energy input and higher biochar yield in comparison to the BPM725 biochar.

As no relationship of the competing metal ions on the sorption efficiency of the selected metal by the investigated biochars was confirmed, the investigated biochars can function as universal sorbents and demonstrate efficiency irrespective of the occurrence of other metals present in the soil environment or water solutions.

In order to improve the sorption capacity of biochars produced from poultry manure, it is justified to subject them to modification for the improvement of specific surface area as well as chemical properties. Our future work will be focused on the improvement of sorption properties of poultry-manure-derived biochars through chemical, physical and thermal modifications.

## Figures and Tables

**Table 1:** Selected properties of poultry manure (PM).

pH _H20_	M, %	Ash, %	TC, %	N, %	P, %
6.5	2.13 ± 0.87	35.28 ± 1.45	38.99 ± 0.910	6.10 ± 0.04	1.61 ± 0.01

M—moisture, TC—total carbon.

**Table 2:** Phases of batch sorption tests.

Process Conditions	Phase I—Mono-Metal in the Solution	Phase II—Combination of Metals in the Solution
Metals present in the solution	Zn Cd Pb	Zn + Cd Zn + Pb Cd + Pb Zn + Cd + Pb
Initial metal concentration in the solution, mg·L^−1^	10, 50, 100, 200, 500, 1000	50, 100, 200
Biochar dose	10 g in 100 mL of the solution (1:10 m/v)	10 g in 100 mL of the solution (1:10 m/v) 5 g in 100 mL of the solution (1:20 m/v)
Exposure time of solid–liquid phase	24 h	24 h
Temperature	22 °C	22 °C
Initial pH of the solution	4	4

**Table 3:** Chemical composition of poultry-manure-derived biochars (selected elements).

Type of Biochar	Ca	Mg	P	K	Cd	Pb	Cr	Cu	Ni	Zn	Hg
	% d.m.				mg·kg^−1^						
BPM425	12.70 ± 0.47	1.32 ± 0.09	3.65 ± 0.01	4.93 ± 0.09	1.04 ± 0.02	8.77 ± 0.13	22.9 ± 0.80	106.0 ± 2.41	21.7 ± 0.43	713.0 ± 2.52	0.0037 ± 0.0
BPM575	16.30 ± 0.34	1.41 ± 0.08	3.28 ± 0.01	4.47 ± 0.104	0.619 ± 0.01	10.20 ± 0.17	20.8 ± 0.81	90.4 ± 1.75	20.5 ± 0.27	639.0 ± 3.05	0.0033 ± 0.0
BPM725	18.10 ± 0.43	1.50 ± 0.01	4.00 ± 0.014	5.55 ± 0.11	<0.300 ± 0.0	9.25 ± 0.36	27.8 ± 0.60	119.0 ± 1.17	30.9 ± 0.42	720.0 ± 4.04	0.0056 ± 0.0

**Table 4:** The elemental analysis of biochars.

Type of Biochar	C	H	N	S	O	H/C	O/C	(N + O)/C
	%					(-)		
BPM425	37.98 ± 0.42	2.16 ± 0.01	4.81 ± 0.01	0.83 ± 0.01	17.31 ± 0.29	0.68	0.34	0.45
BPM575	36.22 ± 0.39	1.01 ± 0.02	2.50 ± 0.05	0.88 ± 0.03	15.67 ± 0.12	0.33	0.32	0.38
BPM725	37.42 ± 0.37	1.00 ± 0.02	2.76 ± 0.07	1.09 ± 0.08	12.99 ± 0.01	0.32	0.26	0.32

**Table 7:** The obtained values of adsorption of single metal ions for the investigated biochars in relation to the initial metal concentration in the solution.

The Initial Concentration of an Ion in the Solution, mg·L^−1^	A, mg·g^−1^			
	Metal	BPM425	BPM575	BPM727
10	Zn	0.0985	0.0996	0.0988
	Cd	0.0999	0.1000	0.1000
	Pb	0.0995	0.0998	0.0992
50	Zn	0.4982	0.4996	0.4988
	Cd	0.4998	0.4999	0.5000
	Pb	0.4983	0.4998	0.4995
100	Zn	0.8964	0.8969	0.8963
	Cd	1.0280	1.0300	1.0300
	Pb	0.8934	0.8952	0.8957
200	Zn	1.9980	1.9996	1.9956
	Cd	1.9969	1.9999	2.0000
	Pb	1.9967	1.9994	1.9994
500	Zn	4.9932	4.9981	4.9794
	Cd	4.9891	4.9995	5.0000
	Pb	4.9923	4.9981	4.9984
1000	Zn	9.9935	9.9972	9.9814
	Cd	9.9793	9.99841	10.0000
	Pb	9.9866	9.9975	9.9972

Not selected SD ˂ 0.01.

**Table 8:** Removal of Zn (%) from the solutions with mono-, double- and triple-metal combinations.

Type of Biochar	Metal Combination in the Solution	Removal, %			Removal, %		
		Biochar Dose: 100 g·L^−1^			Biochar Dose: 50 g·L^−1^		
		50	100	200	50	100	200
BPM425	Zn	99.650 ± 0.017	99.933 ± 0.010	99.902 ± 0.085	99.459 ± 0.116	99.753 ± 0.029	99.718 ± 0.009
BPM575		99.929 ± 0.026	99.994 ± 0.003	99.979 ± 0.003	99.935 ± 0.008	99.882 ± 0.012	99.899 ± 0.013
BPM725		99.757 ± 0.013	99.927 ± 0.009	99.779 ± 0.023	99.910 ± 0.012	99.739 ± 0.034	99.928 ± 0.003
BPM425	Zn + Cd	99.986 ± 0.009	99.919 ± 0.017	99.774 ± 0.062	99.559 ± 0.033	99.620 ± 0.059	99.799 ± 0.079
BPM575		99.920 ± 0.044	99.959 ± 0.027	99.894 ± 0.012	99.992 ± 0.013	99.660 ± 0.425	99.948 ± 0.003
BPM725		99.847 ± 0.063	99.861 ± 0.005	99.853 ± 0.027	99.861 ± 0.058	99.791 ± 0.037	99.821 ± 0.062
BPM425	Zn + Pb	99.916 ± 0.035	99.975 ± 0.010	99.903 ± 0.033	99.501 ± 0.130	99.699 ± 0.027	99.887 ± 0.016
BPM575		99.585 ± 0.012	99.972 ± 0.004	99.846 ± 0.018	99.558 ± 0.575	99.887 ± 0.009	99.913 ± 0.011
BPM725		99.498 ± 0.122	99.690 ± 0.392	99.838 ± 0.051	99.826 ± 0.066	99.836 ± 0.012	99.768 ± 0.063
BPM425	Zn + Cd + Pb	99.890 ± 0.035	99.806 ± 0.021	99.951 ± 0.005	99.419 ± 0.129	99.525 ± 0.121	99.786 ± 0.076
BPM575		99.939 ± 0.034	99.976 ± 0.012	99.964 ± 0.010	99.910 ± 0.029	99.880 ± 0.029	99.920 ± 0.009
BPM725		99.908 ± 0.027	99.899 ± 0.006	99.801 ± 0.031	99.906 ± 0.045	99.796 ± 0.069	99.785 ± 0.046

**Table 9:** Removal of Cd (%) from the solutions with mono-, double- and triple-metal combinations.

Type of Biochar	Metal Combination in the Solution	Removal, %			Removal, %		
		Biochar Dose: 100 g·L^−1^			Biochar Dose: 50 g·L^−1^		
		50	100	200	50	100	200
BPM425	Cd	99.971 ± 0.046	99.808 ± 0.003	99.845 ± 0.013	99.631 ± 0.029	99.570 ± 0.155	99.599 ± 0.054
BPM575		99.999 ± 0.001	100 ± 0	99.999 ± 0.001	99.973 ± 0.021	99.942 ± 0.019	99.897 ± 0.007
BPM725		100 ± 0	100 ± 0	100 ± 0	100 ± 0	100 ± 0	100 ± 0
BPM425	Zn + Cd	99.945 ± 0.025	99.856 ± 0.040	99.708 ± 0.078	99.584 ± 0.118	99.487 ± 0.095	99.644 ± 0.054
BPM575		100 ± 0	99.993 ± 0.003	99.925 ± 0.029	99.995 ± 0.003	99.889 ± 0.012	99.867 ± 0.034
BPM725		100 ± 0	99.998 ± 0.002	100 ± 0	100 ± 0	99.998 ± 0.001	100 ± 0
BPM425	Cd + Pb	99.966 ± 0.015	99.809 ± 0.017	99.851 ± 0.050	99.486 ± 0.096	99.195 ± 0.110	99.392 ± 0.052
BPM575		99.999 ± 0.001	99.994 ± 0.002	99.967 ± 0.002	99.924 ± 0.020	99.857 ± 0.067	99.910 ± 0.013
BPM725		99.999 ± 0.001	100 ± 0	100 ± 0	99.999 ± 0.001	99.998 ± 0.001	100 ± 0
BPM425	Zn + Cd + Pb	99.876 ± 0.019	99.842 ± 0.016	99.800 ± 0.069	99.232 ± 0.118	99.517 ± 0.068	99.722 ± 0.067
BPM575		99.999 ± 0.001	99.971 ± 0.017	99.920 ± 0.016	99.925 ± 0.016	99.894 ± 0.005	99.850 ± 0.043
BPM725		99.999 ± 0.001	99.999 ± 0.001	99.999 ± 0.001	99.998 ± 0	100 ± 0	100 ± 0

**Table 10:** Removal of Pb (%) from the solutions with mono-, double- and triple-metal combinations.

Type of Biochar	Metal Combination in the Solution	Removal, %			Removal, %		
		Biochar Dose: 100 g·L^−1^			Biochar Dose: 50 g·L^−1^		
		50	100	200	50	100	200
BPM425	Pb	99.667 ± 0.181	99.709 ± 0.125	99.837 ± 0.016	99.667 ± 0.031	99.735 ± 0.059	99.812 ± 0.030
BPM575		99.953 ± 0.023	99.906 ± 0.006	99.969 ± 0.009	99.883 ± 0.022	99.717 ± 0.097	99.582 ± 0.009
BPM725		99.899 ± 0.016	99.964 ± 0.010	99.971 ± 0.001	99.950 ± 0.024	99.946 ± 0.011	99.910 ± 0.008
BPM425	Zn + Pb	99.902 ± 0.017	99.735 ± 0.076	99.752 ± 0.098	99.910 ± 0.043	99.630 ± 0.083	99.702 ± 0.035
BPM575		99.949 ± 0.011	99.822 ± 0.060	99.809 ± 0.058	99.717 ± 0.117	99.300 ± 0.142	99.558 ± 0.066
BPM725		99.938 ± 0.017	99.970 ± 0.019	99.963 ± 0.006	99.949 ± 0.024	99.901 ± 0.010	99.903 ± 0.022
BPM425	Cd + Pb	99.828 ± 0.024	99.759 ± 0.043	99.759 ± 0.028	99.651 ± 0.095	99.612 ± 0.064	99.798 ± 0.054
BPM575		99.977 ± 0.015	99.920 ± 0.027	99.920 ± 0.025	99.866 ± 0.021	99.614 ± 0.058	99.678 ± 0.092
BPM725		99.981 ± 0.002	99.963 ± 0.026	99.963 ± 0.001	99.959 ± 0.021	99.918 ± 0.003	99.890 ± 0.146
BPM425	Zn + Cd + Pb	99.914 ± 0.012	99.748 ± 0.107	99.761 ± 0.066	99.513 ± 0.141	99.725 ± 0.043	99.725 ± 0.061
BPM575		99.965 ± 0.003	99.949 ± 0.011	99.819 ± 0.030	99.758 ± 0.043	99.596 ± 0.030	99.596 ± 0.029
BPM725		99.767 ± 0.319	99.943 ± 0.033	99.676 ± 0.046	99.965 ± 0.007	99.946 ± 0.005	99.946 ± 0.021

**Table 11:** Statistical analysis for sorption of Zn (% of removal) in regard to metal combination in the solution and the pyrolysis temperature.

Tested Variable		M	SD	F	*p*	η^2^
Zn		99.84	0.14	0.73	0.537	0.04
Zn + Cd		99.84	0.12			
Zn + Pb		99.78	0.16			
Zn + Cd + Pb		99.84	0.15			
425 °C		99.76	0.17	**5.35**	**0.007**	**0.15**
575 °C		99.89	0.12			
725 °C		99.82	0.09			
Zn	425 °C	99.74	0.17	1.06	0.396	0.10
	575 °C	99.94	0.04			
	725 °C	99.84	0.09			
Zn + Cd	425 °C	99.78	0.17			
	575 °C	99.90	0.12			
	725 °C	99.84	0.03			
Zn + Pb	425 °C	99.81	0.18			
	575 °C	99.79	0.18			
	725 °C	99.74	0.13			
Zn + Cd + Pb	425 °C	99.73	0.21			
	575 °C	99.93	0.04			
	725 °C	99.85	0.06			

M—average; SD—standard deviation; F—the result of the analysis of variance; *p*—significance; η^2^—the size of the effect.

**Table 12:** Statistical analysis for sorption of Cd (% of removal) in regard to metal combination in the solution and the pyrolysis temperature.

Tested Variable		M	SD	F	*p*	η^2^
Cd		99.87	0.26	0.11	0.956	0.01
Zn + Cd		99.88	0.16			
Cd + Pb		99.85	0.24			
Zn + Cd + Pb		99.86	0.20			
425 °C		99.66	0.26	**30.79**	**<0.001**	**0.51**
575 °C		99.95	0.05			
725 °C		100.00	0.00			
Cd	425 °C	99.64	0.36	0.14	0.990	0.01
	575 °C	99.97	0.04			
	725 °C	100.00	0.00			
Zn + Cd	425 °C	99.70	0.17			
	575 °C	99.94	0.06			
	725 °C	100.00	0.00			
Cd + Pb	425 °C	99.62	0.30			
	575 °C	99.94	0.06			
	725 °C	100.00	0.00			
Zn + Cd + Pb	425 °C	99.66	0.25			
	575 °C	99.93	0.05			
	725 °C	100.00	0.00			

M—average; SD—standard deviation; F—the result of the analysis of variance; *p*—significance; η^2^—the size of the effect.

**Table 13:** Statistical analysis for sorption of Pb (% of removal) in regard to metal combination in the solution and the pyrolysis temperature.

Tested Variable		M	SD	F	*p*	η^2^
Pb		99.84	0.13	0.61	0.614	0.03
Zn + Pb		99.80	0.18			
Cd + Pb		99.84	0.13			
Zn + Cd + Pb		99.80	0.14			
425 °C		99.74	0.10	**13.98**	**<0.001**	**0.32**
575 °C		99.78	0.18			
725 °C		99.92	0.07			
Pb	425 °C	99.74	0.07	0.84	0.547	0.08
	575 °C	99.84	0.15			
	725 °C	99.94	0.03			
Zn + Pb	425 °C	99.77	0.11			
	575 °C	99.69	0.23			
	725 °C	99.94	0.03			
Cd + Pb	425 °C	99.73	0.08			
	575 °C	99.83	0.15			
	725 °C	99.95	0.03			
Zn + Cd + Pb	425 °C	99.73	0.13			
	575 °C	99.78	0.16			
	725 °C	99.87	0.12			

M—average; SD—standard deviation; F—the result of the analysis of variance; *p*—significance; η^2^—the size of the effect.

**Table 14:** Adsorption of metal ions in mono-, double- and triple-metal combinations for the investigated biochars.

The Initial Ion Concentration in the Solution mg·L^−1^	Adsorption, mg·g^−1^						
	Ion in Solution	Biochar Dose: 100 g·L^−1^			Biochar Dose: 50 g·L^−1^		
		BPM425	BPM575	BPM725	BPM425	BPM575	BPM725
50	**Zn**	0.4982 ± 0.0001	0.4996 ± 0.0001	0.4988 ± 0.0001	0.9807 ± 0.0009	0.9857 ± 0.0001	0.9851 ± 0.0001
	**Zn** + Cd	0.4449 ± 0.0001	0.4446 ± 0.0002	0.4443 ± 0.0003	0.8861 ± 0.0003	0.8899 ± 0.0001	0.8888 ± 0.0005
	**Zn** + Pb	0.4696 ± 0.0001	0.4680 ± 0.0001	0.4676 ± 0.0006	0.9353 ± 0.0012	0.9358 ± 0.0054	0.9384 ± 0.0006
	**Zn** + Cd + Pb	0.4575 ± 0.0002	0.4577 ± 0.0001	0.4576 ± 0.0001	0.9107 ± 0.0012	0.9152 ± 0.0003	0.9151 ± 0.0004
	**Cd**	0.4998 ± 0.0002	0.4999 ± 0.0001	0.5000 ± 0	1.0561 ± 0.0003	1.0597 ± 0.0002	1.0600 ± 0
	**Cd** + Zn	0.5447 ± 0.0001	0.5450 ± 0	0.5450 ± 0	1.0855 ± 0.0013	1.0899 ± 0.0001	1.0900 ± 0
	**Cd** + Pb	0.5298 ± 0.0001	0.5300 ± 0.0001	0.5300 ± 0.0001	1.0545 ± 0.0010	1.0592 ± 0.0002	1.0600 ± 0.0001
	**Cd** + Zn + Pb	0.5343 ± 0.0001	0.5350 ± 0.0001	0.5350 ± 0.0001	1.0618 ± 0.0013	1.0692 ± 0.0002	1.0700 ± 0
	**Pb**	0.4983 ± 0.0007	0.4998 ± 0.0001	0.4995 ± 0.0001	0.9927 ± 0.0002	0.9948 ± 0.0002	0.9955 ± 0.0002
	**Pb** + Zn	0.4805 ± 0.0001	0.4807 ± 0.0001	0.4807 ± 0.0001	0.9611 ± 0.0004	0.9593 ± 0.0011	0.9615 ± 0.0002
	**Pb** + Cd	0.5041 ± 0.0001	0.5049 ± 0.0001	0.5049 ± 0.0001	1.0064 ± 0.0010	1.0086 ± 0.0002	1.0096 ± 0.0002
	**Pb** + Zn + Cd	0.5145 ± 0.0001	0.5148 ± 0.0001	0.5138 ± 0.0016	1.0250 ± 0.0014	1.0275 ± 0.0004	1.0296 ± 0.0001
100	**Zn**	0.8964 ± 0.0001	0.8969 ± 0.0001	0.8963 ± 0.0001	1.7896 ± 0.0004	1.7919 ± 0.0002	1.7893 ± 0.0005
	**Zn** + Cd	0.9033 ± 0.0001	0.9036 ± 0.0002	0.9027 ± 0.0001	1.8011 ± 0.0011	1.8019 ± 0.0077	1.8042 ± 0.0007
	**Zn** + Pb	0.9058 ± 0.0001	0.9057 ± 0.0001	0.9032 ± 0.0035	1.8065 ± 0.0005	1.8099 ± 0.0002	1.8090 ± 0.0002
	**Zn** + Cd + Pb	0.9471 ± 0.0002	0.9488 ± 0.0001	0.9480 ± 0.0001	1.8890 ± 0.0023	1.8957 ± 0.0005	1.8941 ± 0.0013
	**Cd**	1.0280 ± 0.0001	1.0300 ± 0	1.0300 ± 0	2.0388 ± 0.0032	2.0588 ± 0.0004	2.0600 ± 0
	**Cd** + Zn	1.0784 ± 0.0004	1.0799 ± 0.0001	1.0800 ± 0.0001	2.1489 ± 0.0020	2.1576 ± 0.0002	2.1599 ± 0.0001
	**Cd** + Pb	1.0580 ± 0.0002	1.0599 ± 0.0001	1,0600 ± 0	2.1029 ± 0.0023	2.1170 ± 0.0014	2.1200 ± 0.0001
	**Cd** + Zn + Pb	1.1582 ± 0.0002	1.1597 ± 0.0002	1.1600 ± 0.0001	2.3088 ± 0.0016	2.3175 ± 0.0001	2.3200 ± 0
	**Pb**	0.8934 ± 0.0009	0.8952 ± 0.0001	0.8957 ± 0.0001	1.7873 ± 0.0009	1.7869 ± 0.0014	1.7910 ± 0.0002
	**Pb** + Zn	0.9505 ± 0.0007	0.9513 ± 0.0006	0.9527 ± 0.0002	1.8989 ± 0.0016	1.8927 ± 0.0027	1.9041 ± 0.0002
	**Pb** + Cd	0.9377 ± 0.0004	0.9392 ± 0.0002	0.9396 ± 0.0002	1.8727 ± 0.0012	1.8727 ± 0.0011	1.8785 ± 0.0001
	**Pb** + Zn + Cd	1.0374 ± 0.0011	1.0395 ± 0.0001	1.0394 ± 0.0003	2.0743 ± 0.0009	2.0716 ± 0.0006	2.0789 ± 0.0001
200	**Zn**	1.9980 ± 0.0014	1.9996 ± 0.0001	1.9956 ± 0.0004	3.5699 ± 0.0003	3.5764 ± 0.0004	3.5774 ± 0.0001
	**Zn** + Cd	1.8159 ± 0.0011	1.8181 ± 0.0002	1.8173 ± 0.0005	3.6327 ± 0.0029	3.6381 ± 0.0029	3.6335 ± 0.0022
	**Zn** + Pb	1.8582 ± 0.0006	1.8571 ± 0.0003	1.8569 ± 0.0009	3.7158 ± 0.0006	3.7168 ± 0.0004	3.7114 ± 0.0023
	**Zn** + Cd + Pb	1.8391 ± 0.0001	1.8393 ± 0.0002	1.8363 ± 0.0006	3.6721 ± 0.0028	3.6770 ± 0.0003	3.6721 ± 0.0017
	**Cd**	1.9969 ± 0.0002	1.9999 ± 0.0001	2.0000 ± 0	4.1433 ± 0.0022	4.1557 ± 0.0003	4.1600 ± 0
	**Cd** + Zn	2.1238 ± 0.0016	2.1284 ± 0.0006	2.1300 ± 0	4.2448 ± 0.0023	4.2543 ± 0.0014	4.2600 ± 0
	**Cd** + Pb	2.0669 ± 0.0010	2.0693 ± 0.0001	2.0700 ± 0	4.1148 ± 0.0021	4.1363 ± 0.0005	4.1400 ± 0
	**Cd** + Zn + Pb	2.0459 ± 0.0014	2.0484 ± 0.0003	2.0500 ± 0.0001	4.0886 ± 0.0028	4.0938 ± 0.0017	4.1000 ± 0
	**Pb**	1.9967 ± 0.0003	1.9994 ± 0.0001	1.9994 ± 0.0001	3.7929 ± 0.0009	3.7841 ± 0.0003	3.7966 ± 0.0002
	**Pb** + Zn	1.8953 ± 0.0019	1.8964 ± 0.0011	1.8993 ± 0.0001	3.7887 ± 0.0013	3.7832 ± 0.0025	3.7963 ± 0.0025
	**Pb** + Cd	2.1164 ± 0.0006	2.1177 ± 0.0005	2.1191 ± 0.0001	4.2314 ± 0.0023	4.2263 ± 0.0039	4.2353 ± 0.0062
	**Pb** + Zn + Cd	1.9453 ± 0.0013	1.9465 ± 0.0006	1.9437 ± 0.0009	3.8882 ± 0.0024	3.8929 ± 0.0011	3.8968 ± 0.0008

**Table 15:** The pH values after the completion of sorption from the solutions containing mono-, double- or triple-metal combinations.

Type of Biochar	Combination of Metals in the Solution	pH					
		Biochar Dose: 100 g·L^−1^			Biochar Dose: 50 g·L^−1^		
		50	100	200	50	100	200
BPM425	Zn	9.85	9.76	9.86	10.00	9.75	9.56
BPM575		10.62	10.42	10.46	10.48	10.32	10.15
BPM725		12.63	12.58	12.65	12.40	12.36	12.37
BPM425	Pb	9.88	9.83	9.88	10.01	9.88	9.87
BPM575		11.17	10.51	10.92	10.58	10.50	10.43
BPM725		12.59	12.60	12.57	12.42	12.41	12.41
BPM425	Cd	9.47	9.78	9.72	9.93	9.81	9.76
BPM575		10.69	10.52	10.80	10.57	10.46	10.31
BPM725		12.65	12.60	12.70	12.42	12.42	12.42
BPM425	Zn + Cd	9.66	9.85	9.63	9.86	9.67	9.47
BPM575		10.44	10.34	10.31	10.40	10.20	9.90
BPM725		12.55	12.65	12.56	12.43	12.39	12.37
BPM425	Zn + Pb	9.66	9.82	9.68	9.91	9.69	9.55
BPM575		10.54	10.38	10.32	10.39	10.26	10.09
BPM725		12.59	12.65	12.57	12.44	12.38	12.38
BPM425	Cd + Pb	9.72	9.73	9.70	10.01	9.85	9.65
BPM575		10.55	10.52	10.41	10.51	10.42	10.26
BPM725		12.56	12.65	12.58	12.43	12.40	12.33
BPM425	Zn + Cd + Pb	9.70	9.68	9.59	9.91	9.68	9.34
BPM575		10.48	10.38	10.22	10.42	10.22	9.88
BPM725		12.55	12.65	12.52	12.41	12.38	12.31

## Data Availability

Not applicable.
